# Assessment of the Bacteriological Profile and Antibiotic Susceptibility Patterns of Wastewater in Health Facilities of Ethiopia

**DOI:** 10.1155/2021/9969479

**Published:** 2021-07-15

**Authors:** Belayneh Regasa Dadi, Eyayu Girma, Mheret Tesfaye, Mohamed Seid

**Affiliations:** Department of Medical Microbiology, Arba Minch University, Arba Minch, Ethiopia

## Abstract

**Background:**

Antimicrobials used for the treatment and prevention of bacterial infections are mainly released nonmetabolized into the aquatic environment via wastewater. Sometimes, unused therapeutic drugs are released down the drains that could act as selective pressure for the development of resistance. The aim of this study was to assess the bacteriological profile of wastewater in health facilities and determine antibiotic susceptibility patterns of bacterial isolates.

**Methods:**

A cross-sectional study was conducted from October 1 to December 26, 2020, in health facility wastewater. A total of 128 samples were collected from health facilities for bacteriological analysis and antimicrobial susceptibility testing.

**Result:**

A total of 128 samples were processed, and 81 bacterial isolates were recovered. The most common bacterial isolates were *S. aureus* (16/81 (19.8%)) followed by *Klebsiella* spp. (15/81 (18.5%)), *E. coli* (13/81 (16%)), *P. aeruginosa* (10/81 (12.3%)), *Enterobacter* spp. (8/81 (9.9%)), *Citrobacter* spp. (7/81 (8.6%)), coagulase-negative *Staphylococcus* (5/81 (6.2%)), *Salmonella* spp. (5/81 (6.2%)), and *Shigella* spp. (2/81 (2.5%)). A majority of isolates were resistant to ampicillin (62/81 (76.5%)). Only few isolates were resistant to ciprofloxacin (11/81 (13.6%)), chloramphenicol (13/81 (16%)), and kanamycin (8/54 (14.8%)). A majority of bacterial isolates (57/81 (70.4%)) were multidrug resistant (MDR).

**Conclusion:**

Wastewater from the health facilities contains antibiotic-resistant including multidrug-resistant bacteria. Therefore, health facility wastewater should be treated by appropriate wastewater treatment before being released into the environment.

## 1. Background

Medical waste is categorized into pathological waste—body fluids from surgery, infectious waste from laboratories, pharmaceutical waste—out-of-date pharmaceutical products, and chemical wastes—used solvents, disinfectants, pesticides, and diagnostic chemicals, aerosol containers and gas, and open sources used in in vitro diagnosis or nuclear medical therapy [1]. Sewage from hospitals, usually referred to as hospital waste, is defined as a special category of waste which comprises all wastes, biological or nonbiological, that are discarded from hospitals/healthcare centers and not intended for further use [2]. The important usage of water in hospitals gives significant volumes of waste loaded with microorganisms (the majority of which being pathogenic), heavy metals, toxic chemicals, and radioactive elements [3].

The amount of antibiotics present in hospitals discharge into effluent results, a selection pressure on bacteria [4]. In addition to pharmaceuticals, another type of chemical compound that is heavily used in hospitals and has consequently raised concern about potential environmental toxicity is disinfectants [5]. The discharge of resistant bacteria to the receiving environment can pose public health impacts through carrying the transmissible gene and by acting as a vector or reservoir of the resistant gene [6].

The impact of polluted sewage on the surface and groundwater is widely regarded as a serious threat to human health and environment in many developing countries of the world [7]. In Ethiopia, rapid urbanization and industrialization without deliberating environmental planning often lead to the release of industrial and hospital sewage effluent directly into the environment which is a major problem [8, 9]. The aim of this study was to assess the bacteriological profile of wastewater in health facilities and determine antibiotic susceptibility patterns of bacterial isolates in Arba Minch, Ethiopia, where wastewater from the health facilities is released into the environment without treatment.

## 2. Methods

### 2.1. Sample Collection

A facility-based cross-sectional study was conducted from October to December 2021 at health facilities in Arba Minch, Ethiopia, namely, Arba Minch Hospital, Sikella Health Center, and Secha Health Center.

Each sample was collected four times a day from all sites in 250 mL cleaned and sterile microbiological glass bottles containing 0.2 mL of 3% w/v sodium thiosulphate [10]. The samples were transported within two hours in an ice box to the Microbiology and Parasitology Laboratory of Arba Minch University, College of Medicine and Health Sciences, for analysis.

### 2.2. Sample Size Determination and Sampling Technique

Wastewater samples were collected from the septic tank before being released to the open field according to guidelines of wastewater sampling technique [10]. A total of 128 samples were processed for bacteriological analysis and antimicrobial susceptibility testing.

### 2.3. Microbiological Investigations

The sample was thoroughly shaken to get a homogeneous mixture before a portion was taken for culture. Serial 10-fold dilutions of wastewater samples were prepared in 0.85% NaCl (normal saline). Serial dilution was done in order to get pure colonies. The bacteria were cultivated by plating 0.5 ml of each of the desired serial dilutions of the bacterial suspensions, 7^th^ and 8^th^ (10^−7^and 10^−8^) dilutions of health facility wastewater. Duplicate samples were plated onto MacConkey (MAC) agar, Mannitol Salt Agar (MSA), *Pseudomonas* agar, and Selenite F Broth and then incubated at 37°C for 24–48 hours. After incubation, based on colony morphology, representative colonies were picked and subcultured on different selective and differential media such as blood agar (BA), MacConkey (MAC) agar, Mannitol Salt agar (MSA), Xylose Lysine Deoxycholate (XLD) agar, and *Pseudomonas* agar. After obtaining pure colonies and recording important features, isolated organisms were further identified at the species level by biochemical tests [11].

### 2.4. Antibiotic Susceptibility Testing

The standard Kirby–Bauer disk diffusion method was used to determine the antimicrobial susceptibility profiles of the isolates [12]. Bacterial inocula were prepared by suspending the freshly grown bacteria in 4-5 ml normal saline, and the turbidity was adjusted to 0.5 McFarland standards. Finally, the suspension was streaked onto the entire surface of the Mueller–Hinton agar using a cotton swab to produce confluent growth. Antibiotic discs impregnated with specific amounts of commonly prescribed antimicrobial agents for patient use were then placed on the surface of the medium and aerobically incubated at 37°C for 18–24 hours. The antimicrobial agents used were ceftriaxone (30 *μ*g), ciprofloxacin (5 *μ*g), ampicillin (10 *μ*g), amoxicillin (25 *μ*g), doxycycline (30 *μ*g), gentamycin (10 *μ*g), erythromycin (15 *μ*g), tetracycline (30 *μ*g), chloramphenicol (30 *μ*g), kanamycin (30 *μ*g), and ceftazidime (30 *μ*g). The zones of inhibition were measured, and interpretation was made using the National Committee for Clinical and Laboratory Standards Institute guidelines [13].

### 2.5. Data Quality Assurance

Reagents, culture media, and antimicrobial disks were checked for expiry date, damage, and storage problems. Culture media preparation was made based on the respective manufacturer's instructions. Five percent of media per batch was incubated at 37°C overnight and observed for bacterial growth. Those media which showed growth were discarded. Control strains *Pseudomonas aeruginosa* (ATCC 27853), *Escherichia coli* (ATCC 25922), and *Staphylococcus aureus* (ATCC 25923) were used.

### 2.6. Statistical Analysis

Data were entered, cleaned, and analyzed by using Statistical Package for Social Sciences (SPSS) software version 20.0. One-way ANOVAs, independent Students' *T*-test, and paired *T*-test were used to compare means of some parameters. A critical value of 0.05 was used for the inferential statistics.

## 3. Results

### 3.1. Bacteriological Analysis of Wastewater

A total of 128 samples were processed, and 81 bacterial isolates were recovered. Most bacterial isolates (66/81 (81.5%)) were from Arba Minch Hospital followed by 9/81 (11.1%) bacterial isolates from Sikella Health Center and 6/81 (7.5%) bacterial isolates from Secha Health Center. The most common bacterial isolate was *S. aureus* (16/81 (19.8%)) (Table 1).

### 3.2. Antimicrobial Resistance Patterns of Bacterial Isolates

A majority of isolates were resistant to ampicillin (62/81 (76.5%)). Only few isolates were resistant to ciprofloxacin (11/81 (13.6%)), chloramphenicol (13/81 (16%)), and kanamycin (8/54 (14.8%)).

A majority of *S. aureus* isolates showed resistance to ampicillin (11/16 (68.8%)) and tetracycline (8/16 (50%)), but only few isolates were resistant to ciprofloxacin (3/16 (18.8%)), chloramphenicol (2/16 (12.5%)), ceftazidime (3/16 (18.8%)), and erythromycin (4/16 (25%)). A majority of *Klebsiella* species showed resistance to ampicillin (13/15 (86.7%)), but only few isolates were resistant to chloramphenicol (2/15 (13.3%)), ciprofloxacin (3/15 (20%)), gentamycin (3/15 (20%)), and kanamycin (3/15(20%)). A majority of *E. coli* isolates showed resistance to ampicillin (10/13 (76.9%)), but only few isolates were resistant to gentamycin (1/13 (7.7%)), kanamycin (1/13 (7.7%)), and ciprofloxacin (2/13 (15.4%)). A majority of *Pseudomonas* species showed resistance for ampicillin (8/10 (80%)), but only few isolates were resistant to ciprofloxacin (2/10 (20%)), ceftriaxone (2/10 (20%)), and gentamycin (2/10 (20%)) (see Tables 2–5).

A majority of bacterial isolates from Arba Minch Hospital influent wastewater were resistant to ampicillin (33/40 (82.5%)) and tetracycline (16/35 (46%)) (see Table 2).

Among the isolated bacteria from Arba Minch Hospital effluent wastewater, *Salmonella* species were 100% resistant to ampicillin, coagulase-negative *Staphylococci* were found to be 100% resistant to ceftriaxone, and *Salmonella* species were 50% resistant to ceftriaxone (Table 3).

A majority of bacterial isolates from Sikella Health Center effluent wastewater were resistant to ampicillin (5/9 (55%)). Among bacteria isolates from Sikella Health Center effluent wastewater, *P. aeruginosa* and *Enterobacter* species were found to be 100% resistant to ampicillin, and *Klebsiella* species and *E. coli* were 50% resistant to ampicillin (Table 4).

A majority of bacterial isolates from Secha Health Center effluent wastewater were resistant to ampicillin (5/6(83.3%)). Among bacteria isolates from Secha Health Center effluent wastewater, *Klebsiella* species and *E. coli* were 100% resistant to ampicillin (Table 5).

A majority of bacterial isolates (57/81 (70.4%)) were multidrug resistant (MDR).

## 4. Discussion

In our study, *Staphylococcus aureus* were isolated in high number among wastewater samples from the health facilities continuously released to the receiving environment. Detection of *E. coli* from all sites may be due to the fact that *E. coli* is one of the commensal organisms commonly available in the gastrointestinal tract of humans and animals. Direct and indirect fecal contamination of wastewater from hospital and health centers can easily contaminate the receiving water bodies with potential pathogenic *E. coli* as well as multidrug-resistant strains which can horizontally disseminate to other organisms. The same result was observed in Ethiopia as *E. coli* was detected in high concentration from the effluents of wastewater [14, 15]. Another study done in India showed large numbers of enteric bacteria, and *S. aureus* and *P. aeruginosa* were recovered from wastewater [16]. Similarly, studies done in Thailand [17], Nigeria [18, 19], Tunisia [20], and Ethiopia [15] showed that *Pseudomonas aeruginosa*, *E. coli*, *Staphylococcus aureus*, and *Salmonella* species were the predominant bacteria isolated from health facility wastewater. This may be due to inefficient removal of pathogenic bacteria by oxidation pond or admission of a large number of cases with these bacterial infections [15].

High numbers of bacteria were isolated from health facility wastewater samples. This is an indication of poor and inefficient management of wastewater in community health facilities. The absence of health facility wastewater treatment before releasing wastewater into the sewage system may contribute to the dissemination of such multidrug-resistant bacteria from the health facilities to the environment by draining those bacteria into the city sewage pool or directly into the water bodies such as lakes and rivers. The persistence of large amounts of antibiotics in the environment poses a serious threat to the ecosystem as these could enhance resistance in microbes, which may result in an increase in disease burden along with the change in the structure of the microbial community.

In our study, a majority of bacterial isolates (70.4%) were multidrug resistant (MDR). A similar study conducted in India showed simultaneous resistance of isolates for ampicillin, combination of ampicillin with clavulanic acid, cotrimoxazole, tetracycline, and cephalosporins first, second, and third generation in the final effluent of the wastewater treatment plant [21]. Health facility wastewater contains a diverse group of pathogenic, potentially pathogenic, and environmental bacteria. The characteristic composition makes sewage particularly a suitable environmental condition for the growth and spread of antibiotic resistance due to selection pressure and horizontal gene transfer [22–24].

The present study showed that most bacterial isolates from hospital wastewater show a higher rate of resistance than bacterial isolates from health center wastewater. However, other studies reported that a higher rate of resistance in bacterial isolates from the final effluent of the wastewater treatment plant was found [25, 26]. A similar finding showed that waste effluent from health facilities contains high numbers of drug-resistant bacteria [27].

### 4.1. Limitations of the Study

Although the filter paper is important to filter the microorganisms from the liquid, it helps to easily diagnose the organisms; this study failed to filter the sample due to the lack of the pore membrane.

## 5. Conclusions

High numbers of drug-resistant including multidrug-resistant bacteria were isolated from health facility wastewater samples. Therefore, health facility wastewater should be treated by an appropriate wastewater treatment plant before being released into the environment to minimize dissemination of pathogenic and potentially pathogenic bacteria to the receiving environment.

## Figures and Tables

**Table 1:** Frequency of bacterial isolates from influent and effluent wastewater released from health facilities.

Bacterial isolates	Arba Minch Hospital		Sikella Health Center effluent frequency (%)	Secha Health Center effluent frequency (%)	Total frequency (%)
	Influent frequency (%)	Effluent frequency (%)			
*S. aureus*	7 (17.5)	4 (15.4)	3 (33.3)	2 (33.3)	16 (19.8)
*Klebsiella* spp.	6 (15)	5 (19.2)	2 (22.2)	2 (33.3)	15 (18.5)
*E. coli*	5 (12.5)	4 (15.4)	2 (22.2)	2 (33.3)	13 (16)
*P. aeruginosa*	5 (12.5)	4 (15.4)	1 (11.1)	—	10 (12.3)
*Enterobacter* spp.	5 (12.5)	2 (7.7)	1 (11.1)	—	8 (9.9)
*Citrobacter* spp.	4 (10)	3 (11.5)	—	—	7 (8.6)
Coagulase-negative *Staphylococci*	3 (7.5)	2 (7.7)	—	—	5 (6.2)
*Salmonella* spp.	3 (7.5)	2 (7.7)	—	—	5 (6.2)
*Shigella* spp.	2 (5)	—	—	—	2 (2.5)
Total	40 (100)	26 (100)	9 (100)	6 (100)	81 (100)

**Table 2:** Antibiotic susceptibility patterns of bacterial isolates from influent wastewater released from Arba Minch Hospital.

Bacterial isolates	Antimicrobial agents (frequency (percentage))											
		AMC	AMP	CPR	CTR	CHL	CTX	GN	TET	KAN	DOX	ERY
*P. aeruginosa* [5]	S	—	0 (0)	2 (40)	0 (0)	2 (40)	2 (40)	3 (60)	—	3 (60)	—	—
	I	—	1 (20)	2 (40)	3 (60)	1 (20)	1 (20)	1 (20)	—	0 (0)	—	—
	R	—	4 (80)	1 (20)	2 (40)	2 (40)	2 (40)	1 (20)	—	2 (40)	—	—

*Klebsiella* spp. [6]	S	3 (50)	0 (0)	2 (33)	3 (50)	3 (50)	2 (33)	3 (50)	1 (17)	2 (33)	1 (17)	—
	I	0 (0)	0 (0)	2 (33)	2 (33)	3 (50)	2 (33)	2 (33)	2 (33)	2 (33)	2 (33)	—
	R	3 (50)	6 (100)	2 (33)	1 (17)	0 (0)	2 (33)	1 (17)	3 (50)	2 (33)	3 (50)	—

*E. coli* [5]	S	3 (60)	1 (20)	3 (60)	2 (40)	3 (60)	2 (40)	2 (40)	2 (40)	4 (80)	1 (20)	—
	I	0 (0)	0 (0)	2 (40)	1 (20)	1 (20)	1 (20)	2 (40)	1 (20)	1 (20)	2 (40)	—
	R	2 (40)	4 (80)	0 (0)	2 (40	1 (20)	2 (40)	1 (20)	2 (40)	0 (0)	2 (40)	—

*Citrobacter* spp. [4]	S	3 (75)	0 (0)	2 (50)	2 (50)	2 (50)	2 (50)	2 (50)	2 (50)	2 (50)	2 (50)	—
	I	0 (0)	1 (25)	2 (50)	1 (25)	2 (50)	0 (0)	0 (0)	0 (0)	2 (50)	1 (25)	—
	R	1 (25)	3 (75)	0 (0)	1 (25)	0 (0)	2 (50)	2 (50)	2 (50)	0 (0)	1 (25)	—

*Enterobacter* spp. [5]	S	2 (40)	1 (20)	2 (40)	2 (40)	4 (80)	2 (40)	2 (40)	2 (40)	3 (60)	2 (40)	—
	I	1 (20)	1 (20)	3 (60)	1 (20)	1 (20)	1 (20)	2 (40)	1 (20)	2 (40)	1 (20)	—
	R	2 (40)	3 (60)	0 (0)	2 (40)	0 (0)	2 (40)	1 (20)	2 (40)	0 (0)	2 (40)	—

*S. aureus* [7]	S	2 (29)	1 (20)	3 (43)	4 (57)	6 (72)	3 (43)	4 (57)	0 (0)	—	—	3 (43)
	I	1 (14)	0 (0)	2 (29)	0 (0)	1 (14)	2 (29)	0 (0)	4 (57)	—	—	1 (14)
	R	4 (57)	6 (80)	2 (29)	3 (43)	1 (14)	2 (29)	3 (43)	3 (43)	—	—	3 (43)

Coagulase-negative *Staphylococcus* [3]	S	1 (33)	0 (0)	2 (67)	2 (67)	2 (67)	1 (33)	2 (67)	0 (0)	—	—	1 (33)
	I	0 (0)	0 (0)	1 (33)	1 (33)	1 (33)	1 (33)	1 (33)	2 (67)	—	—	0 (0)
	R	2 (67)	3 (100)	0 (0)	0 (0)	0 (0)	1 (33)	0 (0)	1 (33)	—	—	2 (67)

*Salmonella* spp. [3]	S	3 (100)	0 (0)	2 (67)	1 (33)	2 (67)	1 (33)	2 (67)	0 (0)	—	0 (0)	—
	I	0 (0)	1 (33)	1 (33)	1 (33)	0 (0)	1 (33)	1 (33)	1 (33)	—	1 (33)	—
	R	0 (0)	2 (67)	0 (0)	1 (33)	1 (33)	1 (33)	0 (0)	2 (67)	—	2 (67)	—

AMC: amoxicillin; AMP: ampicillin; CPR: ciprofloxacin; CTR: ceftriaxone; CHL: chloramphenicol; CTX: ceftazidime; GN: gentamycin; TET: tetracycline; KAN: kanamycin; DOX: doxycycline; ERY: erythromycin; S: susceptible; I: intermediate; R: resistant.

**Table 3:** Antibiotic susceptibility patterns of bacterial isolates from effluent released from Arba Minch Hospital.

Bacterial isolates	Antimicrobial agent (frequency (percentage))											
		AMC	AMP	CPR	CTR	CHL	CTX	GN	TET	KAN	DOX	ERY
*P. aeruginosa* [4]	S	—	0 (0)	3 (75)	2 (50)	2 (50)	0 (0)	3 (75)	—	3 (75)	—	—
	I	—	1 (25)	0 (0)	2 (50)	1 (25)	2 (50)	0 (0)	—	0 (0)	—	—
	R	—	3 (75)	1 (25)	0 (0)	1 (25)	2 (50)	1 (25)	—	1 (25)	—	—

*Klebsiella* spp. [5]	S	2 (40)	1 (20)	3 (75)	2 (40)	3 (60)	1 (20)	3 (60)	2 (40)	3 (60)	1 (20)	—
	I	1 (20)	0 (0)	1 (20)	0 (0)	0 (0)	4 (80)	0 (0)	1 (20)	1 (20)	2 (40)	—
	R	2 (40)	4 (80)	1 (20)	3 (60)	2 (40)	0 (0)	2 (40)	2 (40)	1 (20)	2 (40)	—

*E. coli* [4]	S	2 (50)	0 (0)	2 (50)	3 (75)	3 (75)	2 (50)	1 (25)	2 (50)	2 (50)	2 (50)	—
	I	1 (25)	1 (25)	1 (25)	1 (25)	0 (0)	0 (0)	3 (75)	1 (25)	1 (25)	0 (0)	—
	R	1 (25)	3 (75)	1 (25)	0 (0)	1 (25)	2 (50)	0 (0)	1 (25)	1 (25)	2 (50)	—

*Citrobacter* spp. [3]	S	2 (67)	0 (0)	2 (67)	1 (33)	2 (67)	0 (0)	2 (67)	2 (67)	2 (67)	1 (33)	—
	I	0 (0)	1 (33)	1 (33)	1 (33)	1 (33)	1 (33)	1 (33)	0 (0)	0 (0)	1 (33)	—
	R	1 (33)	2 (67)	0 (0)	1 (33)	0 (0)	2 (67)	0 (0)	1 (33)	1 (33)	1 (33)	—

*Enterobacter* spp. [2]	S	1 (50)	1 (50)	1 (50)	2 (100)	1 (50)	2 (100)	2 (100)	0 (0)	1 (50)	2 (100)	—
	I	0 (0)	0 (0)	0 (0)	0 (0)	1 (50)	0 (0)	0 (0)	1 (50)	1 (50)	0 (0)	—
	R	1 (50)	1 (50)	1 (50)	0 (0)	0 (0)	0 (0)	0 (0)	1 (50)	0 (0)	0 (0)	—

*S. aureus* [4]	S	2 (50)	1 (25)	2 (50)	2 (50)	2 (50)	1 (25)	3 (75)	0 (0)	—	—	2 (50)
	I	1 (25)	0 (0)	1 (25)	0 (0)	1 (25)	2 (50)	0 (0)	1 (25)	—	—	1 (25)
	R	1 (25)	3 (75)	1 (25)	2 (50)	1 (25)	1 (25)	1 (25)	3 (75)	—	—	1 (25)

CONS [2]	S	1 (50)	0 (0)	1 (50)	0 (0)	1 (50)	2 (100)	1 (50)	1 (50)	—	—	1 (50)
	I	0 (0)	1 (50)	1 (50)	0 (0)	0 (0)	0 (0)	0 (0)	1 (50)	—	—	0 (0)
	R	1 (50)	1 (50)	0 (0)	2 (100)	1 (50)	0 (0)	1 (50)	0 (0)	—	—	1 (50)

*Salmonella* spp. [2]	S	1 (50)	0 (0)	2 (100)	0 (0)	1 (50)	0 (0)	1 (50)	1 (50)	—	0 (0)	—
	I	0 (0)	0 (0)	0 (0)	1 (50)	0 (0)	1 (50)	1 (50)	0 (0)	—	1 (50)	—
	R	1 (50)	2 (100)	0 (0)	1 (50)	1 (50)	1 (50)	0 (0)	1 (50)	—	1 (50)	—

AMC: amoxicillin; AMP: ampicillin; CPR: ciprofloxacin; CTR: ceftriaxone; CHL: chloramphenicol; CTX: ceftazidime; GN: gentamycin; TET: tetracycline; KAN: kanamycin; DOX: doxycycline; ERY: erythromycin; S: susceptible; I: intermediate; R: resistant.

**Table 4:** Antibiotic susceptibility patterns of bacterial isolates from effluent released from Sikella Health Center.

Bacterial isolates	Antimicrobial agents (frequency (percentage))											
		AMC	AMP	CPR	CTR	CHL	CTX	GN	TET	KAN	DOX	ERY
*P. aeruginosa* [1]	S	—	0 (0)	1 (100)	1 (100)	1 (100)	1 (100)	1 (100)	—	1 (100)	—	—
	I	—	0 (0)	0 (0)	0 (0)	0 (0)	0 (0)	0 (0)	—	0 (0)	—	—
	R	—	1 (100)	0 (0)	0 (0)	0 (0)	0 (0)	0 (0)	—	0 (0)	—	—

*Klebsiella* spp. [2]	S	2 (100)	0 (0)	2 (100)	2 (100)	2 (100)	2 (100)	2 (100)	2 (100)	2 (100)	2 (100)	—
	I	0 (0)	1 (50)	0 (0)	0 (0)	0 (0)	0 (0)	0 (0)	0 (0)	0 (0)	0 (0)	—
	R	0 (0)	1 (50)	0 (0)	0 (0)	0 (0)	0 (0)	0 (0)	0 (0)	0 (0)	0 (0)	—

*E. coli* [2]	S	2 (100)	1 (50)	1 (50)	2 (100)	1 (50)	1 (50)	2 (100)	2 (100)	2 (100)	2 (100)	—
	I	0 (0)	0 (0)	0 (0)	0 (0)	0 (0)	1 (50)	0 (0)	0 (0)	0 (0)	0 (0)	—
	R	0 (0)	1 (50)	1 (50)	0 (0)	1 (50)	0 (0)	0 (0)	0 (0)	0 (0)	0 (0)	—

*Enterobacter* spp. [1]	S	1 (100)	0 (0)	1 (100)	1 (100)	1 (100)	1 (100)	1 (100)	1 (100)	1 (100)	1 (100)	—
	I	0 (0)	0 (0)	0 (0)	0 (0)	0 (0)	0 (0)	0 (0)	0 (0)	0 (0)	0 (0)	—
	R	0 (0)	1 (100)	0 (0)	0 (0)	0 (0)	0 (0)	0 (0)	0 (0)	0 (0)	0 (0)	—

*S. aureus* [3]	S	1 (33)	1 (33)	2 (66)	3 (100)	2 (67)	3 (100)	1 (33)	2 (67)	—	—	3 (100)
	I	1 (33)	1 (33)	1 (33)	0 (0)	1 (33)	0 (0)	2 (67)	0 (0)	—	—	0 (0)
	R	1 (33)	1 (33)	0 (0)	0 (0)	0 (0)	0 (0)	0 (0)	1 (33)	—	—	0 (0)

AMC: amoxicillin; AMP: ampicillin; CPR: ciprofloxacin; CTR: ceftriaxone; CHL: chloramphenicol; CTX: ceftazidime; GN: gentamycin; TET: tetracycline; KAN: kanamycin; DOX: doxycycline; ERY: erythromycin; S: susceptible; I: intermediate; R: resistant.

**Table 5:** Antibiotic susceptibility patterns of bacterial isolates from effluent released from Secha Health Center.

Bacterial isolates	Antimicrobial agents (frequency (percentage))											
		AMC	AMP	CPR	CTR	CHL	CTX	GN	TET	KAN	DOX	ERY
*Klebsiella* spp. [2]	S	2 (100)	0 (0)	2 (100)	2 (100)	2 (100)	2 (100)	2 (100)	2 (100)	2 (100)	2 (100)	—
	I	0 (0)	0 (0)	0 (0)	0 (0)	0 (0)	0 (0)	0 (0)	0 (0)	0 (0)	0 (0)	—
	R	0 (0)	2 (100)	0 (0)	0 (0)	0 (0)	0 (0)	0 (0)	0 (0)	0 (0)	0 (0)	—

*E. coli* [2]	S	2 (100)	0 (0)	1 (50)	2 (100)	2 (100)	2 (100)	2 (100)	2 (100)	2 (100)	1 (50)	—
	I	0 (0)	0 (0)	1 (50)	0 (0)	0 (0)	0 (0)	0 (0)	0 (0)	0 (0)	0 (0)	—
	R	0 (0)	2 (100)	0 (0)	0 (0)	0 (0)	0 (0)	0 (0)	0 (0)	0 (0)	1 (50)	—

*S. aureus* [2]	S	2 (100)	0 (0)	2 (100)	1 (50)	2 (100)	1 (50)	2 (100)	1 (50)	—	—	2 (100)
	I	0 (0)	1 (50)	0 (0)	1 (50)	0 (0)	1 (50)	0 (0)	0 (0)	—	—	0 (0)
	R	0 (0)	1 (50)	0 (0)	0 (0)	0 (0)	0 (0)	0 (0)	1 (50)	—	—	0 (0)

AMC: amoxicillin; AMP: ampicillin; CPR: ciprofloxacin; CTR: ceftriaxone; CHL: chloramphenicol; CTX: ceftazidime; GN: gentamycin; TET: tetracycline; KAN: kanamycin; DOX: doxycycline; ERY: erythromycin; S: susceptible; I: intermediate; R: resistant.

## Data Availability

The datasets generated and/or analyzed during the current study are not publicly available due to ethical and confidentiality reasons but are available from the corresponding author upon reasonable request under the ethics committee's approval.
